# The 21st Century Cures Act: Inpatient Clinician Perceptions of Changes to Information Sharing at an Academic Medical Center

**DOI:** 10.7759/cureus.40184

**Published:** 2023-06-09

**Authors:** Mohamad K Hamze, Shubhankar S Joshi, Yao Li, Allen B Repp, Alicia Jacobs, Rachel McEntee

**Affiliations:** 1 Department of Medicine, The Robert Larner, M.D. College of Medicine at the University of Vermont, Burlington, USA; 2 Department of Medicine, University of Vermont Medical Center, Burlington, USA; 3 Department of Family Medicine, The Robert Larner, M.D. College of Medicine at the University of Vermont, Burlington, USA; 4 Department of Family Medicine, University of Vermont Medical Center, Burlington, USA

**Keywords:** 21st century cures act, medical documentation, cures act, access to information, inpatient care, academic medical centers, personal health information, electronic health record (ehr), information sharing

## Abstract

Introduction

To comply with the Information Blocking Rule in the 21st Century Cures Act, many hospitals began to release inpatient electronic health information such as clinical notes and results to patients immediately, starting in April 2021. We sought to understand the perceptions of hospital-based clinicians regarding the impact of these changes in information sharing on clinicians and patients.

Materials and methods

We developed and distributed an electronic survey to 122 inpatient attending physicians, resident physicians, and physician assistants within the internal medicine and family medicine departments at an academic medical center. The survey asked clinicians to rate their comfort with information-sharing protocols and describe their perceptions of the impact of immediate information sharing on their documentation habits and patient interactions following the implementation of the Cures Act.

Results

The survey response rate was 37.7% (46/122). Of the respondents, 56.5% felt comfortable with the note-sharing process, 84.8% reported omitting specific information from their notes to prevent patients from reading it, and 39.1% of clinicians agreed that patients have found clinical notes “more confusing than helpful.”

Conclusions

Immediate sharing of electronic health information has the potential to be a powerful tool for communicating with hospitalized patients. However, our results show many hospital-based clinicians report limited comfort with the note-sharing process and perceive it to be confusing to patients. Efforts are needed to educate clinicians regarding information sharing, understand patient and family perspectives, and develop best practices to enhance communication through electronic notes.

## Introduction

Implementation of the Cures Act

The 21st Century Cures Act (“Cures Act”) is a federal law, one component of which mandates the immediate release of inpatient and outpatient progress notes, laboratory test results, and radiology and pathology reports to the patient’s electronic health record (EHR) portal [1]. The Information Blocking Rule (“the Rule”) within the Cures Act took effect on April 5, 2021, and established a core set of electronic health information (EHI) via the United States Core Data for Interoperability (USCDI) that must be made available to patients in a timely fashion [2-4]. Although many studies have assessed the impact of information-sharing changes on clinicians and patients in the outpatient setting [5,6], few studies have investigated clinician perceptions of EHI sharing in the inpatient setting. As part of a project to improve the quality of communication in the EHR at our medical center, we developed and conducted a survey to assess clinician understanding and perceptions of immediate EHI sharing as a result of the Rule.

Information-sharing changes from the Cures Act

As of April 5, 2021, the Rule prohibits healthcare providers from engaging in practices that are “unreasonable and likely to interfere with access, exchange or use of EHI” unless certain limited exceptions apply [4]. In essence, the Rule prevents providers from delaying or restricting patients’ access to their EHI, including results, progress notes, and pathology and radiology reports. The guidelines of the Rule state that all health systems must share the required EHI with patients in both inpatient and outpatient settings. From June 2017 until April 2021, our institution was sharing all patient notes written by providers in ambulatory encounters but only discharge summaries from inpatient hospital visits. On April 5, 2021, our institution began the immediate release of inpatient progress notes, imaging narratives, laboratory and pathology results, and consult notes. Notes and other patient information that are part of the USCDI may only be withheld from the patient if an exception permitted by the regulations applies. The most clinically relevant exceptions are those circumstances that pose a specific risk to patient safety or privacy [7].

Challenges of information sharing in the inpatient setting

While the goal of the Cures Act and information sharing is to provide transparency to patients and families regarding their medical care, many clinicians have noted apprehension and concerns about the default sharing of patient notes [5]. Clinicians have expressed worry that notes, and results may contain information that could be confusing, unexpected, or distressing without accompanying explanation by the patient care team. As a result, it is possible that clinicians may change the content of clinical documentation or omit information altogether [8]. Prior studies have examined clinician and patient perspectives in the inpatient setting, but only when notes were being released at the time of discharge [9]. This project sought to explore whether these concerns were present among inpatient clinicians at our institution when notes and results were being released in real time.

This article was previously presented in the form of a virtual poster presentation at the 2021 American College of Physicians Vermont Chapter Annual Scientific Meeting on October 15, 2021.

## Materials and methods

Institution-wide emails were distributed to clinicians at our institution ahead of the April 5, 2021 implementation of the Information Blocking Rule to inform clinicians about the workflow and protocols regarding information sharing and blocking changes. We conducted an anonymous, web-based survey of resident physicians, attending physicians, and physician assistants in the internal medicine and family medicine specialties within our institution in July 2021, three months following the implementation of these changes. Survey participants were clinicians who provided care to hospitalized patients after the implementation of the Information Blocking Rule on April 5, 2021. This survey was developed using the Likert scale, yes/no, and open-response answer formats where applicable. This instrument was partly modeled on a 2018 survey composed by DesRoches and colleagues, which aimed to assess clinician experiences with note sharing before the implementation of the Cures Act rules [5]. Our survey included 23 rating questions in addition to a demographics section. The survey in its entirety can be found in the Appendix.

Email invitations were sent out to 122 inpatient clinicians (45 internal medicine and family medicine MD/DO (doctor of medicine/doctor of osteopathic medicine) attending physicians or physician assistants, and 77 internal medicine and family medicine resident physicians) between July 14, 2021, and August 3, 2021. As an incentive to complete the initial survey, clinicians could elect to provide their contact information for the chance to win a $10 Amazon e-gift card. Study data were collected and managed using REDCap (Vanderbilt University, Nashville, TN) electronic data capture tools hosted at our institution.

According to the policy defining research activity at our institution, this work met the criteria for operational quality improvement activities exempt from IRB review.

## Results

Immediate changes in note sharing following the Cures Act implementation 

**Figure 1:**
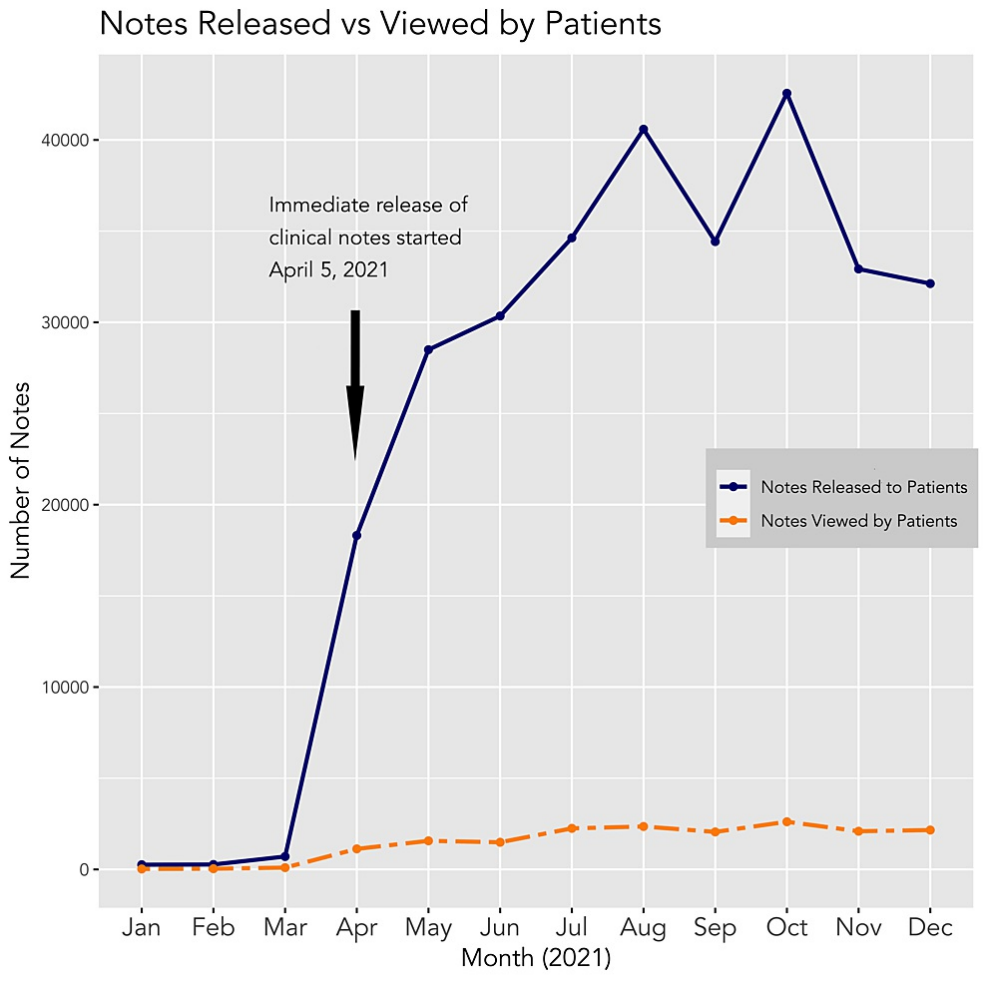
The number of inpatient notes shared and viewed in 2021. Following the implementation of the Information Blocking Rule on April 5, 2021, the absolute number of inpatient notes released to and viewed by patients increased at this institution while the percentage of viewed notes decreased Data source: Epic MyChart Data.

The absolute number of notes shared for inpatients increased 26-fold, from 708 in March 2021 to 18,304 in April 2021 following the implementation of the Information Blocking Rule on April 5, 2021. The total number of shared notes per month in the calendar year 2021 reached a maximum of 42,561 in October. The absolute number of notes viewed by patients increased 11-fold from 99 in March 2021 to 1,126 in April 2021 following the implementation of the Information Blocking Rule, and to a maximum of 2,613 in October 2021. Figure 1 shows the number of notes released to and viewed by patients in 2021.

Survey demographics

Survey respondent demographics are presented in Table 1. The mean age of the respondents was 34 years (SD = 8.9).

**Table 1:** Demographics of survey respondents

Attribute		Respondents	Percentage (%)
Sample size		122	100
Respondents		46	37.7
Training level	Resident physician	26	56.5
	Attending physician	18	39.1
	Physician assistant	2	4.3
Gender	Male	25	54.3
	Female	21	45.7

Clinician familiarity and comfort with information sharing

**Figure 2:**
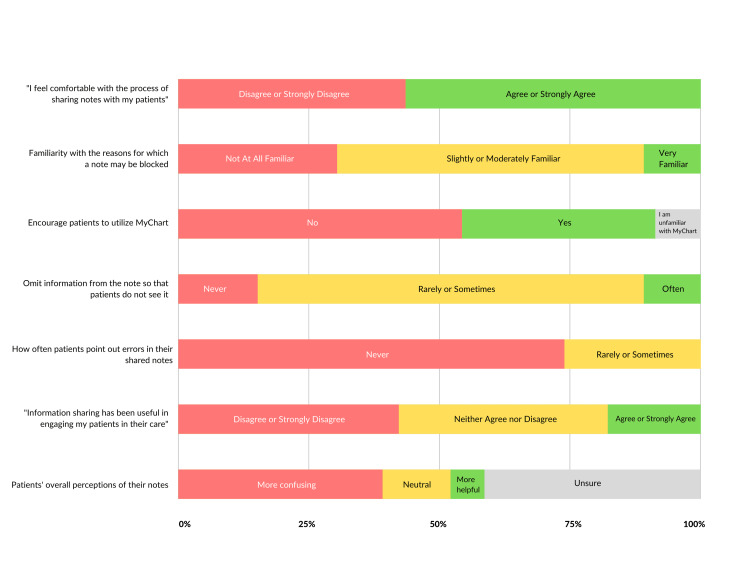
Responses to selected survey items

Responses to selected questions are presented in Figure 2. Most respondents (93.5%, 43/46) were aware that the Information Blocking Rule went into effect in the inpatient setting at our institution on April 5, 2021, while 37% (17/46) endorsed encouraging their patients to utilize the MyChart electronic patient portal to access their inpatient notes (Figure 2). Just over half of all respondents (56.5%, 26/46) agreed or strongly agreed they felt comfortable with the current note-sharing process. Regarding familiarity with the note-withholding process, 30.4% (14/46) of respondents felt “not at all familiar” with the reasons for which they could withhold a note from being viewed by their patients.

Note writing changes

Most respondents (65.2%, 30/46) reported that the time spent writing their notes did not change after the implementation of the Rule; however, 28.3% (13/46) reported at least some increase in time spent composing notes. Most respondents (84.8%, 35/46) indicated that they omitted specific information or language from their notes to prevent their patients from reading it, with 10.8% (5/46) indicating that they omitted information “often.”

Patient correspondence regarding notes

Regarding communication with patients, 28.2% (13/46) of respondents noticed at least some increase in time spent conversing with patients or families via phone, email, or the patient portal. When asked how often patients reported errors in their shared notes, 73.9% (34/46) of responding clinicians indicated “Never.” The remainder (26.1%, 12/46) described this as “rarely” (8) or “sometimes” (4). Among the 12 clinicians who responded that patients reported errors “rarely” or “sometimes,” 11 stated that patients reported errors in the history/subjective portion of the chart, while five stated that patients reported errors in medications, and one stated that a patient reported an error in the physical exam.

Clinician perceptions of patient experience with notes

When surveyed about how they felt patients perceived their notes, 39.1% (18/46) of clinicians felt their patients found the notes “more confusing than helpful.” Since the implementation of the Rule, 41.3% (19/46) of clinicians noted their patients “expressed more worry” and 34.8% (16/46) noted that patients “expressed more confusion.” None noted that their patients “expressed more reassurance,” “complied better with instructions and recommendations,” or “understood their discharge instructions better.”

Clinician experience with notes

When asked if they anticipated any challenges or barriers to the digital storage, review, and sharing of clinical notes, 52.2% (24/46) of responding clinicians reported that they anticipated ethical barriers to information sharing, including those related to privacy. Several clinicians wrote responses about their experience with patients or patient families reading what they perceived to be sensitive information before the clinician could counsel them on the result in question. Overall, 42.2% (19/45) either disagreed or disagreed strongly with the general statement: “Information sharing through the Cures Act has been useful in further engaging my patients with their care.”

Site-specific physician training with information sharing

Most survey respondents (86.7%, 40/46) felt they were at least slightly aware of the changes to information sharing at our institution, and 54.3% (25/46) endorsed knowing how to withhold inpatient notes from being shared through the patient portal if it met one of the allowed exceptions to the Rule. Over half (54.3%, 25/46) of the respondents indicated they would be interested in further training in the process of information sharing, with 18 of these 25 indicating that they would prefer this training in the form of a written reference/tip sheet. Other training options suggested by survey respondents were asynchronous video training modules or on-site, one-on-one training, although these were less popular.

## Discussion

Key findings

Though it is reported in the literature that patients want immediate and full access to their EHI [7], fewer than one in four clinicians perceived that information sharing has been useful in engaging patients in their inpatient care, and many clinicians indicated concern that notes may be confusing to patients. These findings highlight the potential disconnect between hospitalized patients and inpatient clinicians regarding the use of information sharing as a tool in their care. In addition, more than a third of respondents reported that their patients expressed worry or confusion about the content of their shared notes.

The majority of surveyed clinicians endorsed omitting certain information or language from their notes as a result of the implementation of the Information Blocking Rule. Although this study did not explore the content or type of omissions, it is possible that clinicians omitted clinical assessments or interpretations that they felt might be confusing or worrisome to patients. Alternatively, clinicians may have avoided language to describe patients or their conditions, which has the potential to, intentionally or unintentionally, elicit bias or perpetuate stigma [10]. Note writers may also be omitting jargon or abbreviations that would make it difficult for patients to understand their notes. Future research could aim to assess how these omissions affect the perceived utility of notes for patients and the care team.

About one in four respondents reported that patients identified errors in their medical records. This finding raises the possibility that information sharing may increase the accuracy of the medical record, and by extension, reduce medical and diagnostic errors, which may result from inaccurate information, although medical and diagnostic errors were not specifically reported by our respondents. Future investigations might explore the association between EHI sharing, information accuracy, and medical errors.

The absolute number of inpatient notes shared to and viewed by patients increased substantially following the implementation of the Information Blocking Rule, although the overall proportion of shared notes that were viewed by patients remained low, typically under 10%. This finding could have a number of explanations, such as patients not being aware of the note availability in the patient portal, lack of a device to access the portal, lack of patient interest in reading notes, or selective review of notes. Further study is warranted to evaluate the reasons why patients are not accessing notes more frequently.

Limitations

The study’s generalizability is limited by the sample size and focus on two specialties (internal medicine and family medicine) at a single medical center. Clinicians at other medical centers or of other specialties may have varying experiences with information sharing due to different patient populations and the complexity of medical data. Although our response rate was relatively low at 37.7%, raising the possibility of non-response bias, this rate is comparable to, if not better than that of 35% as described by Cunningham and colleagues in a 2015 review exploring physician response rates to online surveys [11]. The Likert scale questions utilized in the survey are subjective by nature, and nominal answer choices may be interpreted variably by different respondents. For example, the answer choice “often” may mean once a week to one respondent and once a day to another. As a whole, although tested and refined, our survey instrument has not been rigorously validated.

Future aims and research directions

Overall, our results indicate that inpatient clinicians have concerns about the utility of automatic EHI sharing. With the results from this project, we have developed several recommendations for areas of focus regarding information sharing. Primarily, it will be critical to assess and understand patient experiences with and perspectives of inpatient EHI sharing, including its usefulness, clarity, and accessibility. This could be accomplished by future surveys of or interviews with previously hospitalized patients. We also are implementing EHR functionality that will allow clinicians to see when patients have read shared notes, which may give clinicians a better understanding of what notes or results their patients have seen and help guide future discussions with patients or families. An easily accessible reference sheet could help clinicians feel more confident with the process of note sharing and withholding. Development and dissemination of best practices for clinicians to communicate with patients about EHI sharing may promote effective information transfer, alleviate clinician discomfort, improve patient understanding and engagement, and enhance care outcomes.

## Conclusions

While previous studies of information sharing show that patients desire access to their EHR notes, few studies thus far have investigated clinician perceptions of EHI sharing in the hospital setting. Following the implementation of note sharing for hospitalized patients at our academic medical center, many inpatient clinicians at our institution perceived that note sharing was not useful in engaging hospitalized patients in their care and may lead to patient confusion. They also endorsed the desire for additional training and professional education on maintaining compliance with new information-sharing rules. Further studies of patient perspectives and the development of evidence-based practices to promote effective EHI sharing are needed.

## Figures and Tables

**Figure 3:**
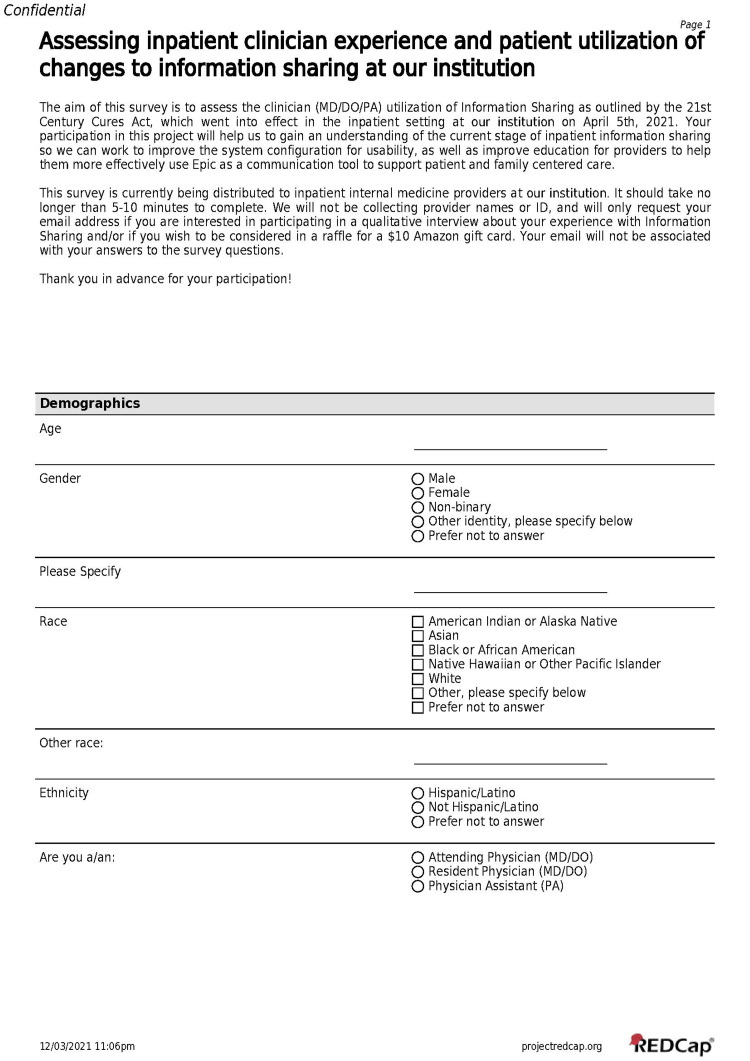
Page 1 of the clinician survey

**Figure 4:**
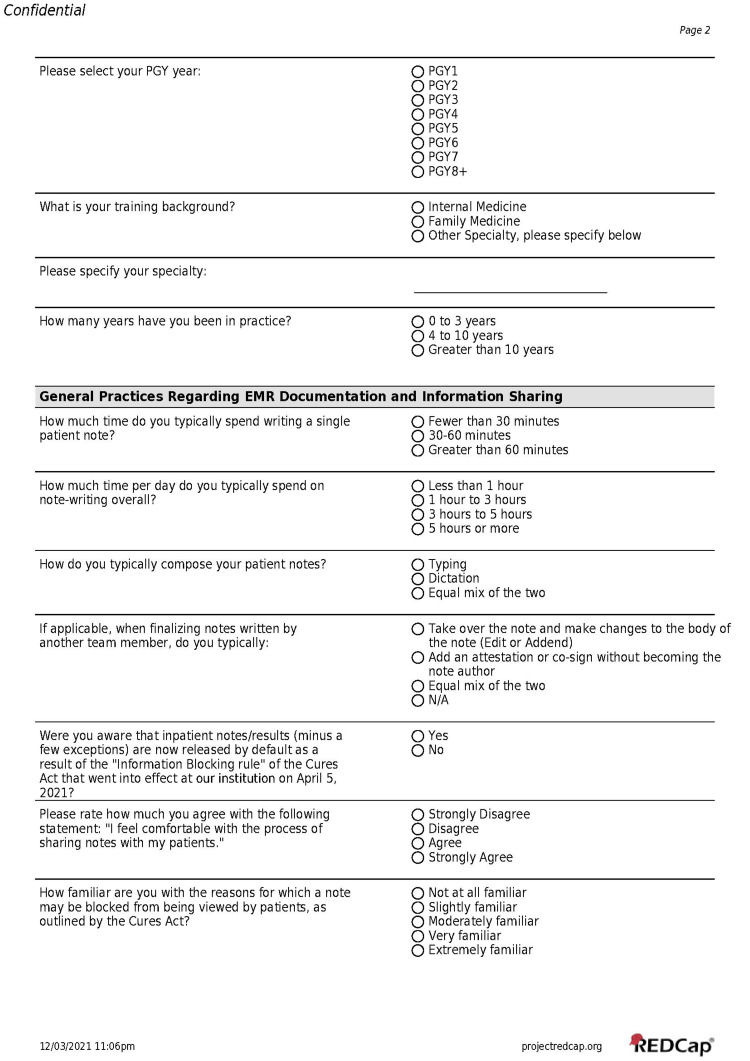
Page 2 of the clinician survey

**Figure 5:**
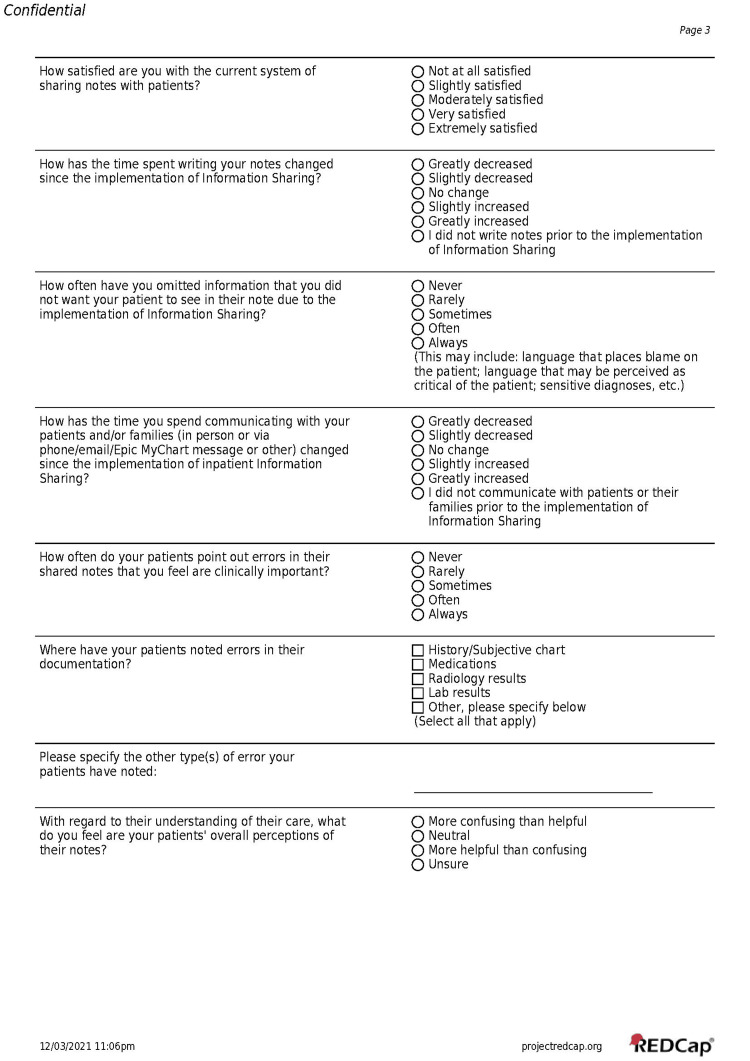
Page 3 of the clinician survey

**Figure 6:**
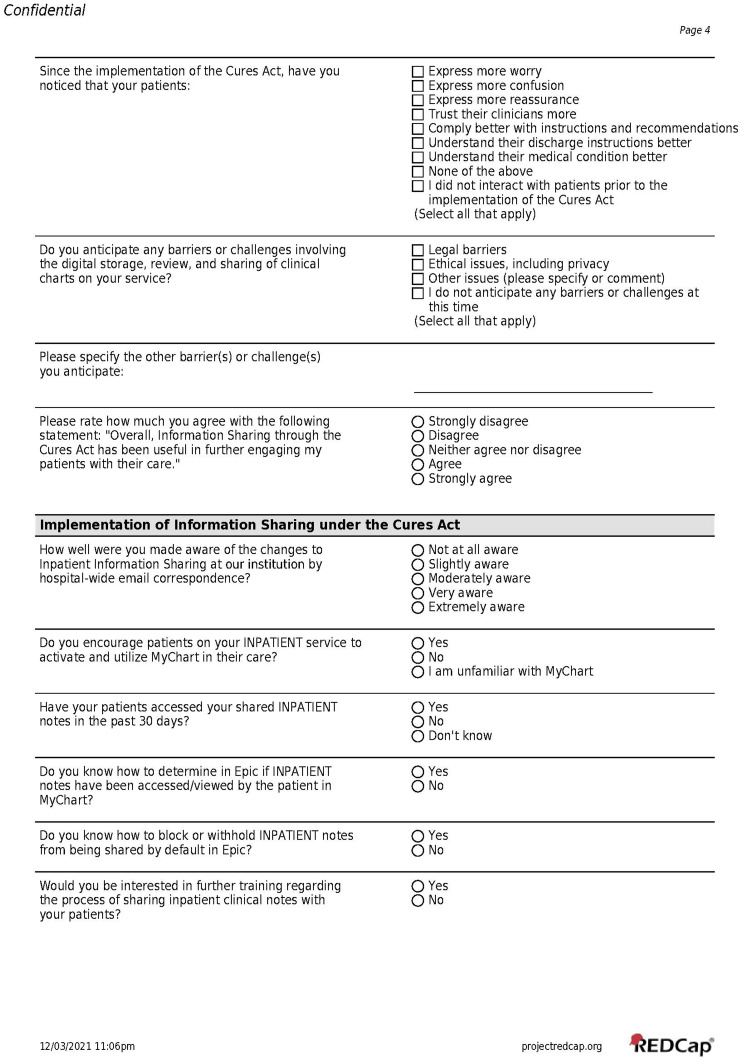
Page 4 of the clinician survey

**Figure 7:**
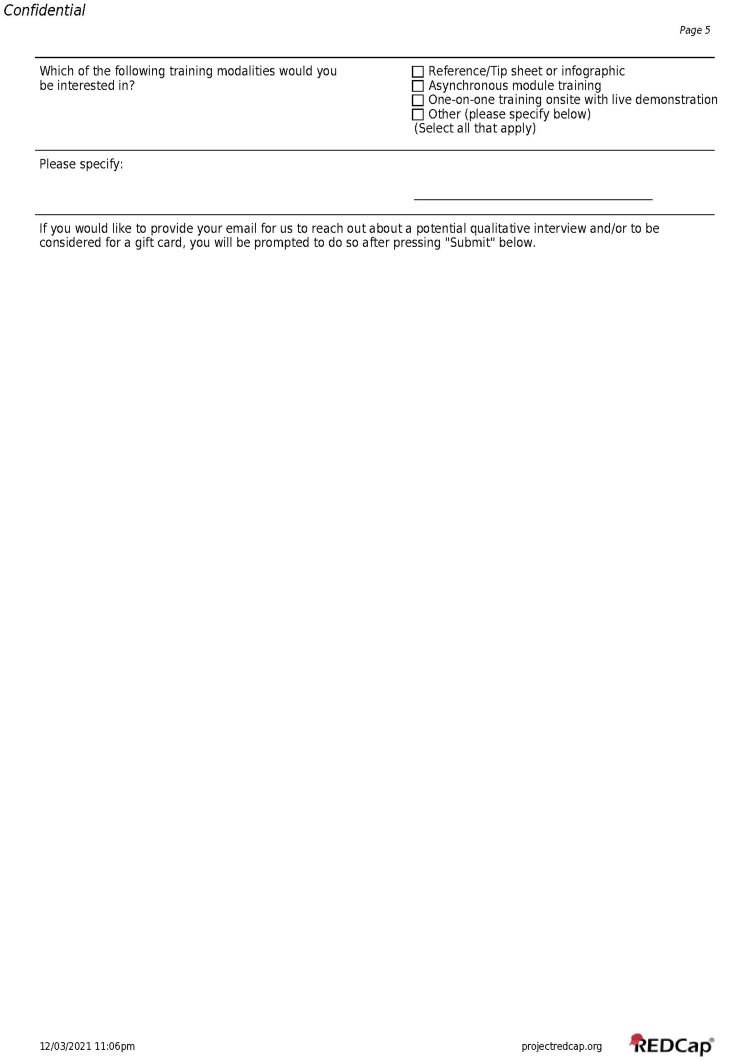
Page 5 of the clinician survey

## Appendices

Appendix
